# Do environmental effects indexed by parental genetic variation influence common psychiatric symptoms in childhood?

**DOI:** 10.1038/s41398-023-02348-y

**Published:** 2023-03-18

**Authors:** Eshim S. Jami, Anke R. Hammerschlag, Hannah M. Sallis, Zhen Qiao, Ole A. Andreassen, Per M. Magnus, Pål R. Njølstad, Alexandra Havdahl, Jean-Baptiste Pingault, David M. Evans, Marcus R. Munafò, Eivind Ystrom, Meike Bartels, Christel Middeldorp

**Affiliations:** 1 Department of Biological Psychology, Vrije Universiteit Amsterdam, Amsterdam, the Netherlands; 2 Department of Clinical, Educational and Health Psychology, University College London, London, UK; 3 School of Psychological Science, University of Bristol, Bristol, UK; 4 MRC Integrative Epidemiology Unit, University of Bristol, Bristol, UK; 5 Centre for Academic Mental Health, Population Health Sciences, University of Bristol, Bristol, UK; 6 The University of Queensland Diamantina Institute, The University of Queensland, Brisbane, Australia; 7 NORMENT Centre, Division of Mental Health and Addiction, Oslo University Hospital & Institute of Clinical Medicine, University of Oslo, Oslo, Norway; 8 KG Jebsen Centre for Neurodevelopment, Institute of Clinical Medicine, University of Oslo, Oslo, Norway; 9 Centre of Fertility and Health, Norwegian Institute of Public Health, Oslo, Norway; 10 Center for Diabetes Research, Department of Clinical Science, University of Bergen, Bergen, Norway; 11 Children and Youth Clinic, Haukeland University Hospital, Bergen, Norway; 12 Department of Mental Disorders, Norwegian Institute of Public Health, Oslo, Norway; 13 PROMENTA Research Center, Department of Psychology, University of Oslo, Oslo, Norway; 14 Nic Waals Institute, Lovisenberg Diakonale Hospital, Oslo, Norway; 15 Medical Research Council Integrative Epidemiology Unit at the University of Bristol, Bristol, UK; 16 Population Health Sciences, Bristol Medical School, University of Bristol, Bristol, UK; 17 NIHR Biomedical Research Centre at the University Hospitals Bristol NHS Foundation Trust and the University of Bristol, Bristol, UK; 18 Child Health Research Centre, University of Queensland, Brisbane, Australia; 19 Child and Youth Mental Health Service, Children’s Health Queensland Hospital and Health Service, Brisbane, Australia

**Keywords:** Psychology, Genetics

## Abstract

Parental genes may indirectly influence offspring psychiatric outcomes through the environment that parents create for their children. These indirect genetic effects, also known as genetic nurture, could explain individual differences in common internalising and externalising psychiatric symptoms during childhood. Advanced statistical genetic methods leverage data from families to estimate the overall contribution of parental genetic nurture effects. This study included up to 10,499 children, 5990 mother–child pairs, and 6,222 father–child pairs from the Norwegian Mother Father and Child Study. Genome-based restricted maximum likelihood (GREML) models were applied using software packages GCTA and M-GCTA to estimate variance in maternally reported depressive, disruptive, and attention-deficit hyperactivity disorder (ADHD) symptoms in 8-year-olds that was explained by direct offspring genetic effects and maternal or paternal genetic nurture. There was no strong evidence of genetic nurture in this sample, although a suggestive paternal genetic nurture effect on offspring depressive symptoms (variance explained (V) = 0.098, standard error (SE) = 0.057) and a suggestive maternal genetic nurture effect on ADHD symptoms (V = 0.084, SE = 0.058) was observed. The results indicate that parental genetic nurture effects could be of some relevance in explaining individual differences in childhood psychiatric symptoms. However, robustly estimating their contribution is a challenge for researchers given the current paucity of large-scale samples of genotyped families with information on childhood psychiatric outcomes.

## Introduction

Psychiatric symptoms in children are often linked to parental characteristics [1–5], but these intergenerational associations are not necessarily causal with the parental characteristics having a direct effect on the offspring symptoms. As parents and children share both genes and environment, parent-offspring associations may be explained by genetic as well as environmental factors [6]. Insight into genetic versus environmental mechanisms of intergenerational transmission and their respective impact on individual differences in children’s psychiatric traits is important to provide families and healthcare providers with adequate information about the aetiology of children’s symptoms and for the development of interventions targeting modifiable factors.

The most common types of psychiatric difficulties experienced during childhood are inward-focused internalising traits characterised by negative affect, such as depression or anxiety, as well as outward-focused externalising traits such as disruptive behaviours or attention-deficit hyperactivity disorder (ADHD) symptoms characterised by poor impulse control or inattention [7]. Evidence from twin studies, which help to differentiate the contribution of genetic and environmental influences to variation in traits, shows that large proportions of variance (40–80%) in these internalising and externalising traits during childhood is explained by genetic factors [8, 9]. This suggests that genetic transmission is a key factor in explaining associations between parental characteristics and these childhood psychiatric symptoms. Twin studies also provide some hints on parental contributions through the environment. For instance, the shared environment, reflecting all aspects of the common environment experienced by children in the same family (including parental effects), explains a proportion of individual differences in internalising and externalising traits in primary school aged children [10, 11]. However, this effect may include other (i.e., non-parental) aspects of the environment and additionally, parental factors may also influence children through the environment that is not shared between siblings. This challenge in assessing the overall importance of parental contributions can now be addressed through the use of novel family-based genetic designs.

Family-based genetic designs can leverage DNA variation from parents and children to study the overall impact of heritable parental traits on an offspring phenotype. This effect, in which parental genetic variation may influence offspring outcomes through the environment that parents create for their children, is referred to as an indirect genetic effect or *genetic nurture* [12, 13]. Current family-based genetic designs use the most common type of DNA sequence variation, known as single nucleotide polymorphisms (SNPs), to study the overall impact of genetic nurture on an offspring trait by modelling the cumulative effect of millions of parental SNPs on offspring behaviour [14–16]. This technique does not involve the study of measured parental behaviours, but examines the effect of parental genetic variants as a proxy for the parentally provided environment. Designs that estimate the overall effect of genetic nurture in this way include molecular genetics technique known as genome-based restricted maximum likelihood (GREML) estimation. The standard GREML approach estimates variance in a given trait that trait that is explained by common genetic variation (i.e., SNP-based heritability). This is done by exploring whether unrelated individuals that are more similar genetically also show more phenotypic similarity [17]. Extended GREML designs include both offspring and parental genotype in the same model to estimate direct and indirect genetic effects on a phenotype [14–16]. In other words, this analysis allows the partition of variance into the effect of the offspring’s genotype (direct genetic effect), the effect of the parent’s genotype (indirect genetic effect, i.e., genetic nurture), and the effect of the covariance between the two. The covariance term reflects the contribution of genes present in both the parent and offspring. This effect corresponds to a passive gene-environment correlation, when parents pass on both trait-associated genes and environment to the child [18]. Hence, extended GREML models also offer a way of estimating the contribution of at least a part of the gene-environment correlation that explains individual differences in offspring traits.

Given the recent development and the data requirements of these types of analyses, the application of GREML models to study genetic nurture effects on common childhood psychiatric symptoms is scarce, with only three publications to date. Our previous work used an extended GREML method called maternal-effects genome-wide complex trait analysis (M-GCTA) [15] to separately model maternal and paternal genetic nurture effects on depressive and anxiety symptoms in 8-year-olds from the Norwegian Mother Father and Child cohort study (MoBa), a population-based sample with an extensive, and growing, number of genotyped families. We found non-significant but suggestive maternal and paternal genetic nurturing effects on offspring depressive, but not anxiety, symptoms [19]. Two subsequent studies performed in the same cohort used other GREML methods (described in detail elsewhere) [15, 16] to estimate parental effects on childhood internalising traits [20] and externalising traits [21]. These studies, based on a newer MoBa data release with additional participants, found evidence of combined parental effects on childhood depressive symptoms, as well as inattention, hyperactivity and conduct problems, but not anxiety or oppositional defiant behaviour [20, 21]. The latter study did not detect meaningful distinctions between maternal and paternal effects on childhood externalising traits using trio-GCTA [21], while the earlier study could not distinguish between maternal and paternal effects on internalising traits using the Relatedness Disequilibrium Regression (RDR) approach [20]. This highlights a key difference between extended GREML methods, in that RDR only estimates a combined parental effect, while M-GCTA and trio-GCTA can model individual parental effects, either in separate maternal and paternal models (M-GCTA) or a joint model (trio-GCTA) [15, 16, 22].

The current study uses the M-GCTA approach to investigate maternal and paternal genetic nurture effects on common childhood psychiatric symptoms. We consider maternal and paternal effects separately as there may be differences in parental effects depending on the sex of the parent. An advantage of the M-GCTA approach is that it can estimate individual parental effects while carrying a lower burden for sample size requirements by estimating fewer parameters in a single model than trio-GCTA. We extend our previous work by examining direct and indirect genetic effects on externalising traits, in addition to internalising symptoms, in a larger dataset of genotyped families in MoBa. More specifically, we focus on depressive, disruptive, and ADHD symptoms which represent the most common psychiatric disorders experienced during childhood [7]. As no strong evidence of genetic nurture effects on anxiety has been indicated in previous literature [19, 20], we excluded anxiety from this study. We discuss and compare the results of our M-GCTA models in relation to other GREML-based methods and provide recommendations for future research estimating genetic nurture effects on childhood psychiatric traits.

## Methods

### Sample

The Norwegian Mother, Father and Child Cohort Study (MoBa) is a population-based pregnancy cohort study conducted by the Norwegian Institute of Public Health. Pregnant women were recruited from all over Norway from 1999-2008 [23]. The women consented to participation in 41% of the pregnancies. The cohort now includes 114,500 children, 95,200 mothers and 75,200 fathers [24]. After birth, information on offspring outcomes was gathered through parental questionnaires at regular follow-up intervals. Parent and infant DNA samples were collected at birth and stored in a biobank [25]. Of these, genotyped data from 98,110 individuals comprised of ~33,000 trios (mothers, fathers and offspring) is currently available in MoBa Genetics. The current study is restricted to a subset of this sample consisting of 14,064 unique individuals in the offspring generation, for whom data on psychiatric symptoms was also available. This was linked to parental genotype data from 13,690 mothers and 13,299 fathers.

The establishment and data collection in MoBa is based on regulations related to the Norwegian Health Registry Act. The current study was approved by The Regional Committee for Medical Research Ethics (REK 2013/863) and is based on version 11 of the quality-assured data files released for research in 2018.

### Measures

The outcome measures were maternally rated depressive, disruptive, and ADHD symptoms in 8-year-olds. Depressive symptoms were measured using the parent version of the Short Mood and Feelings Questionnaire (SMFQ) [26]. The 13-item scale is based on DSM-III-R criteria for depression, and consists of a series of descriptive phrases regarding the child’s recent feelings and behaviours that were rated on a 3-point Likert scale. Example items include ‘felt miserable or unhappy’ and ‘didn’t enjoy anything at all’. The externalising traits (disruptive and ADHD symptoms) were measured using the Parent/Teacher Rating Scale for Disruptive Behaviour Disorder (RS-DBD) which relates to DSM-III-R criteria for oppositional defiant disorder, conduct disorder, and ADHD [27]. As conduct disorder and oppositional defiant disorder are both disruptive disorders that are characterised by children acting out against other children or adults through defiant behaviours, we combined the oppositional defiant and conduct disorder subscales of the RS-DBD, consisting of 8 items each, to measure overall disruptive symptoms. Example items include ‘deliberately annoys people’ and ‘argues with adults’ for the oppositional defiant subscale and ‘bullies, threatens or intimidates others’ and ‘initiates physical fights’ for the conduct disorder subscale. The attention-deficit hyperactivity disorder (ADHD) subscale of the RS-DBD, consisting of 18 items, was used to measure ADHD symptoms, characterised by inattention and hyperactivity. Example items include ‘has difficulty sustaining attention in tasks or play activities’ and ‘talks excessively’. All RSD-BD items were rated on a 4-point Likert scales. Childhood depressive, disruptive, and ADHD symptom scores were calculated with maximum allowed missingness of two items for the SMFQ, three items for the RD-DBD disruptive scale and four items for the RS-DBD ADHD subscale. Missing items were imputed with the mean of the non-missing responses.

### Genotyping

The current release of the MoBa Genetics dataset consists of ~33,000 trios who were genotyped as part of a collaborative research effort, consisting of four major research projects. Genotyping, quality control and imputation procedures were performed separately for each subproject according to standard practices and are described in detail elsewhere (https://github.com/folkehelseinstituttet/mobagen). After imputation of missing genotypes, all datasets were merged to create the MoBa Genetics dataset. Using this dataset as the starting point, we conducted post-imputation quality control to select high-quality SNPs for analysis. SNPs were selected if they met the following standard criteria: Hardy-Weinberg equilibrium *p* < 1 × 10^−6^, 90% genotyping rate, minor allele frequency > 0.05, high imputation quality (INFO score > 0.9 on average across batches), non-multiallelic, and non-duplicated. 5.1 million SNPs were retained for subsequent analysis. All analyses were performed on the core ethnic sample, consisting of individuals of European ancestry who form 95% of the study population with genotypes currently available [28].

### Statistical analyses

To first obtain estimates of the variance in childhood psychiatric symptoms explained by offspring genotype without correcting for parental genotypes (offspring model), the GCTA software package was used [17]. A genomic relatedness matrix (GRM) was constructed to index genetic similarity between the 14,064 genotyped offspring in the dataset. Based on this GRM, a correlation cut-off threshold of 0.025 was applied to exclude excessive relatedness, as the presence of closely related individuals can bias variance estimates. This resulted in a reduced sample size of up to 10,499 individuals. A GREML model was run in GCTA to estimate variance in offspring depressive, disruptive and ADHD symptoms explained by offspring genetic variants (V_*o*_).

The M-GCTA software package [22] implements extended GREML models to estimate variance in offspring phenotype that is explained by direct offspring genetic effects, genetic nurture and gene-environment correlation. As M-GCTA estimates maternal and paternal effects in separate models, the overall genotyped dataset was first split into separate mother–child and father–child datasets using Plink 1.96 [29]. Using the mother–child dataset, M-GCTA was then used to construct multiple GRMs indexing genetic similarity between: (1) individuals within the offspring generation, (2) individuals within the maternal generation, and (3) unrelated mother-child pairs (i.e., offspring from X family and mother from Y family). The same, but for fathers, was repeated for individuals in the father–child dataset. A correlation cut-off threshold of 0.025 was applied using each of the constructed GRMs to exclude excessive relatedness within the offspring generation, the parental generation, and between unrelated pairs across the generations in the mother–child and father–child datasets. After this step, 5,990 pairs in the mother–child and 6222 pairs in the father–child dataset were retained.

Using the mother–child dataset (maternal model), extended GREML analyses were carried out in M-GCTA to estimate the proportion of variance in childhood depressive, disruptive and ADHD symptoms that was explained by offspring genotype (V_o_; corrected for maternal genetic nurture effect), maternal genotype (V_*m*_ i.e., maternal genetic nurture) and the covariance between offspring and maternal genotypes (V_*om*_ i.e., passive gene-environment correlation between offspring genetic effects and maternal genetic nurture). To test for significance, the full model was compared to a model that only estimated the effect of offspring genotype. The analyses were repeated using the father–child dataset (paternal model) to estimate the variance explained by offspring genotype (V_o_; corrected for paternal genetic nurture effect), paternal genotype (V_*f*_ i.e., paternal genetic nurture) and the covariance between offspring and paternal genotypes (V_*of*_ i.e., passive gene-environment correlation between offspring genetic effects and paternal genetic nurture).

The following covariates were regressed out of the outcomes in all analyses: child sex (to account for differences between males and females in the average scores of the outcome phenotypes), genotyping batch and ten genetic principal components based on offspring genotype (to correct for population structure).

## Results

The overall sample of children had a mean age of 8.08 with a standard deviation (SD) of 0.67. There were slightly more boys (52%) than girls in this study. The mean SMFQ score for depressive symptoms was 14.85 (SD = 2.45) and the scores ranged from 13 to 36. The mean RS-DBD score for disruptive symptoms was 20.28 (SD = 4.28) and the range was from 16 to 53. The mean score RS-DBD score for ADHD symptoms was 26.72 (SD = 7.61) and the range was from 18 to 72. Sex differences in all scales were observed, with boys scoring slightly higher than girls (*p* = 0.004 for depressive symptoms, *p* < 0.001 for disruptive and ADHD symptoms). Moderate correlations between symptom scores were observed; depressive symptoms were correlated with disruptive symptoms (*r* = 0.52, *p* < 0.001) and ADHD symptoms (*r* = 0.53, *p* < 0.001), which in turn were also correlated with each other (*r* = 0.59, *p* < 0.001).

### GREML models

Full results for the offspring, maternal and paternal GREML models are presented in Table 1.

**Table 1:** Estimates of variance explained, standard errors, and sample sizes of the fitted models.

	V_*o*_ (SE)	V_*m/f*_ (SE)	V_om/of_ (SE)	G (SE)	logL	*p*	*N*
**Depressive symptoms**							
Offspring model	0.053 (0.027)	–	–	0.053 (0.027)	−5250.08	0.021	10475
Maternal model	0.002 (0.057)	0.029 (0.060)	0.076 (0.045)	0.107 (0.064)	−2828.09	0.312	5964
Paternal model	0.074 (0.059)	0.098 (0.057)	0.000 (0.044)	0.172 (0.062)	−2932.84	0.181	6184
**Disruptive symptoms**							
Offspring model	0.029 (0.026)	–	–	0.030 (0.027)	−5111.40	0.128	10493
Maternal model	0.029 (0.060)	0.041 (0.060)	0.000 (0.047)	0.070 (0.064)	−2750.72	0.263	5966
Paternal model	0.087 (0.061)	0.019 (0.058)	0.001 (0.047)	0.108 (0.063)	−2718.52	0.369	6196
**ADHD symptoms**							
Offspring model	0.101 (0.029)	–	–	0.102 (0.029)	−5215.83	<0.001	10499
Maternal model	0.063 (0.060)	0.084 (0.058)	0.000 (0.048)	0.155 (0.065)	−2737.43	0.069	5972
Paternal model	0.000 (0.056)	0.000 (0.058)	0.038 (0.045)	0.038 (0.060)	−2833.18	0.500	6204

*V*_*o*_ = variance explained by offspring genetic effects, *V*_*m/f*_ = variance explained by maternal or paternal genetic nurture, *V*_*om/of*_ = variance explained by a passive gene-environment correlation between offspring genetic effects and maternal or paternal genetic nurture, *G* = variance explained by combined direct and indirect genetic effects, *SE* = standard error, *logL* = log-likelihood value, *p* = *p*-value, *N* = sample size.

Note: the offspring model estimating standard SNP-based heritability was implemented in GCTA, and maternal and paternal models were implemented in M-GCTA.

The offspring models estimated variance in childhood psychiatric symptoms explained by the child’s own genotype, without correcting for parental genotype. In these models, offspring genetic effects explained 5% of variance in their depressive symptoms (95% confidence interval (CI) = 0.008–11%), 3% of variance in their disruptive symptoms (95% CI = −2–8%), and 10% of variance in ADHD symptoms (95% CI = 4–16%).

The maternal and paternal models estimated variance in childhood psychiatric symptoms explained by direct genetic effects, maternal or paternal genetic nurture, and the covariance between offspring and maternal or paternal genetic effects. After correcting for maternal or paternal genetic nurture effects, variance explained by direct effects of offspring’s own genetic effects varied, with inconsistent estimates and wider confidence intervals (Table 1; maternal and paternal models). There was no strong evidence of maternal or paternal genetic nurture effects on childhood psychiatric behaviours. However, estimates for paternal genetic nurture effects on offspring depressive symptoms (10%, 95% CI = −1–21%) and maternal genetic nurture effects on offspring ADHD symptoms (8%, 95% % CI = −3–20%) were higher than others and could point to suggestive genetic nurture effects that might be different from zero with more power.

As genetic nurture effects were not robust, with large confidence intervals overlapping zero, we cannot meaningfully interpret the observed covariances between offspring genetic effects and parental genetic nurture estimates.

## Discussion

This study utilised parent and offspring genotypic data to estimate maternal and paternal genetic nurture effects on depressive, disruptive, and ADHD symptoms in 8-year-olds within a large population-based Norwegian sample. Although no strong evidence of genetic nurture emerged, there was some indication of a paternal genetic nurture effect on offspring depressive symptoms and a maternal genetic nurture effect on ADHD symptoms which could be different from zero with more power.

We estimated maternal and paternal genetic nurture effects on childhood psychiatric symptoms using the M-GCTA approach. To gauge whether our findings are likely to reflect genuine genetic nurture effects or be spurious, it is useful to compare our results to existing publications using M-GCTA and other GREML methods to investigate genetic nurture effects on childhood internalising and externalising traits in the MoBa cohort. In doing so, we first note that despite using the same dataset we retained fewer participants in our models than comparable studies due to a more stringent threshold for relatedness in our methodology. This reduction in power is reflected in our results. For depression, findings from the current and previous M-GCTA analyses did not identify robust evidence of genetic nurture effects, although small maternal and/or paternal effects were suggested [19]. With a less stringent exclusion criteria, a parental effect was robustly estimated by Cheesman et al. using the RDR approach, and was found to be partly mediated by maternal anxiety and depressive symptoms [20]. Altogether, it is likely that maternally driven genetic nurture effects explain at least a part of the variance in children’s depressive symptoms in the MoBa cohort, but might go undetected when the analysis is based on a smaller subsample with lower power. Findings for paternally driven effects were more inconsistent and further investigation in better-powered analyses is needed to clarify the presence and importance of these effects.

For externalising traits, our results do not directly replicate the recent findings of Eilertsen et al. who used the trio-GCTA method, but are somewhat in line with their findings. For instance, our results indicated a suggestive maternal effect on childhood ADHD symptoms, while the trio-GCTA study identified robust parental effects on both inattention and hyperactivity symptoms of ADHD [21]. Additionally, where we found no strong evidence of genetic nurture effects on disruptive symptoms, the trio-GCTA study found robust parental effects on conduct, but not oppositional defiant behaviours. It is plausible that indirect genetic effects might be of differential importance for subtypes of disruptive behaviours and are masked when conduct and oppositional defiant behaviours are grouped together. If validated, this could inform research on environmentally mediated influences on disruptive behaviours and be a factor of consideration in whether these effects on distinct disruptive subtypes should be studied independently or as a higher-order phenotype. Finally, our results provide some indication that maternal effects might explain more variation in externalising traits than paternal effects. This trend was also observed in the trio-GCTA study of externalising subtypes [21], although meaningful distinctions between parental effects could not be made in either study. Exploring differences in effects between mothers and fathers remains an important avenue for future work.

Estimates of children’s own genetic effects on depressive and ADHD symptoms in the offspring models are in line with previous literature, showing SNP heritability estimates of between 0–17% for depressive and 0–34% for ADHD symptoms [30]. Similarly, the low estimate of SNP heritability for disruptive symptoms (3%; non-significant) is within the wide range (0–54%) observed in previous studies [30], but in particular matches the estimate from the recent GWAS of childhood aggression (3%) [31]. The considerable range within estimates of SNP heritability can be explained by factors including the method, sample size, selection of SNPs, and genomic relatedness threshold, all of which may have an impact on the estimation of variance components using genetic data. It should be noted that these estimates of SNP heritability in the offspring models could include potential indirect genetic effects from relatives and the inclusion of family data in GREML analyses is necessary for identifying if this is the case.

This study adds to a small body of work investigating whether genetic nurture effects are of importance in the development of psychiatric symptoms during childhood [19–21]. It is important to note that this type of research is still in its infancy stages. More effort to clarify the presence, impact and underlying mechanisms of these effects is needed to understand to what extent potential effects encapsulate specific parental factors that could be targeted for intervention, or reflect other influences, such as assortative mating or population stratification [32]. Additionally, as all current findings are based on 8-year-olds of European ancestry in a Norwegian cohort, it is unknown to what extent these effects are present in other populations and at other developmental periods. For example, differences in the importance of genetic nurture effects on childhood educational achievement between Dutch and British samples were observed in a recent study [33]. Given that environmental influences on mental health may vary across communities and developmental stages, it is plausible that potential indirect genetic effects could be of differential importance in different contexts. More investment in global datasets with genotyped parents and children who also have information on childhood psychiatric phenotypes will provide opportunities for examining the role of parental effects across different cultures, societies, and contexts.

The study and design have other limitations that should be acknowledged. First, power is a considerable concern for extended GREML analyses. Power calculations suggest that as many as 50,000 genotyped pairs could be required to estimate maternal and offspring genetic effects with a realistic effect size [34]. Given the paucity of large-scale family-based datasets, collaborative efforts that combine data from multiple cohorts is of importance. Identifying techniques to maximise power in family-based GREML studies is also necessary. One approach could be to set less stringent thresholds for exclusions based on relatedness than were used in the current study. Second, while the M-GCTA method estimates maternal and paternal effects in separate models, recent work indicates that modelling maternal and paternal effects together will provide more accurate estimates of offspring and parental genetic effects [35]. In subsequent research, researchers may want to give trio-GCTA preference over M-GCTA, although it carries a higher sample size burden due to additional parameters. Finally, as our analyses are based on maternally reported psychiatric symptoms, it is possible that the estimates in this study could be impacted by responder bias [36, 37], if for instance mothers with higher genetic susceptibility to psychiatric difficulties report higher symptoms in their offspring. This could be addressed by incorporating information from other informants, e.g., fathers and teachers, in future work.

This study used GREML models to estimate variance in childhood depressive, disruptive and ADHD symptoms that could be explained by common genetic variance in children and their parents. While there was some suggestive indication of parental genetic nurture effects, follow-up analyses in better-powered samples are required to obtain more reliable effect estimates. If robust genetic nurture effects on childhood psychiatric symptoms are identified, the subsequent step in this line of research would be to identify mediating factors that account for these effects and investigate whether they could be targeted for intervention.
